# Supplementary material for: The importance of cognitive diversity for sustaining the commons
Source: Nat Commun. 2019 Feb 20;10:875. doi: 10.1038/s41467-019-08549-8 (PMC6382855; doi:10.1038/s41467-019-08549-8)
Supplement: Supplementary file 1 — Supplementary Information [file 41467_2019_8549_MOESM1_ESM.pdf]

The importance of cognitive diversity for sustaining the commons

Baggio et al.

January 10, 2019

1 Supplementary Note 1

1.1 g and ToM

General intelligence g and theory of mind ToM are considered different but related concepts. Both g and ToM are necessary in order to solve complex problems both at the individual level and within groups. g and ToM relate to functionally different cognitive abilities.

g was measured via ACT scores. The ACT is a college admission test that is widely used in the U.S.A. The ACT correlates (although only moderately) with college GPA, and strongly with SAT (another test widely used in college admissions throughout the U.S.A. [1]).

ToM was measured using the Short Story Test (SST) as proposed by Dodell-Feder and colleagues [2]. The SST allows one to elicit if and how well participants infer the thoughts and emotions of characters in a short story. In other words, the SST estimates the ability of individuals to infer others' intentions and feelings and thus relates to social-cognitive theory of mind [2]. The SST is often used to predict social communications disorders, communication errors, and inferring mental states of others [2].

SST (our measure of ToM) relates to social-cognitive processes and thus should be related to g. However, in our experimental study we only find a weak correlation between ToM and g as reported below.

	$min(ToM)$	$avg(ToM)$	$avg(g)$	$min(g)$
$min(ToM)$	1.0000			
$avg(ToM)$	0.7927	1.0000		
$avg(g)$	0.2930	0.2908	1.0000	
$min(g)$	0.3158	0.2356	0.7960	1.0000

1.2 Ostrom Institutional Design Principles

Hardin's seminal paper on the tragedy of the commons [3] prescribed strong state control or the establishment of private property rights as the only two ways to avoid resource depletion in the case of common pool resources (i.e., resources shared by a community). Since then, however, numerous studies have shown that under specific conditions resource users are able to maintain resources sustainably [4, 5, 6, 7, 8, 9]. Generally speaking, communities with high trust and reciprocity, as well as the ability to understand resource dynamics, are able to devise rules and norms that can favor collective action and reduce or prevent the depletion of resources [6]. The ability of communities to build such rules and norms is enhanced by the presence of specific characteristics or institutional design principles [6, 9, 7]. More specifically, eight principles are put forth by Ostrom and re-classified by Cox and colleagues [9]:

1. Boundaries: Clearly defined boundaries both around the community of users (who has rights to withdraw/harvest common resources) **and** the resource itself (i.e. an irrigation system, a forest, fishery ground etc.).

2. Congruence: There should be congruence between rules of harvest and local resource conditions, **and** there should be proportionality between how much one can harvest from the common resources and how much one invest or help providing that very same resource (working maintaining canals, fish gear, forest patches etc.).

3. Collective choice arrangement: Ability for community members to participate and modify the rules that govern the common resources.

4. Monitoring: Communities should be able to monitor behavior of those who have access to the common resources **and** monitor the monitors.

5. Graduate sanctions: Sanctions should be proportionate to the severity and repetition of rule violations

6. Conflict resolution: Communities should able to solve (at low cost) conflicts that may arise within the community, especially in relation to the appropriation of common resources

7. Minimum rights recognition: communities should be able to effectively create their own institutions (rules and norms) without being continuously challenged by higher hierarchical authorities

8. Nestedness: Governance activities should be organized in different layers from local to regional to state to international.

These design principles indeed increase the likelihood that communities successfully and sustainably manage resources (with a few exceptions) [7, 8]. All these design principles relate to the ability of communities to devise rules and norms that aid in the sustainable management of resources. The ability of groups to devise such institutional principles is underpinned by their ability to effectively negotiate and communicate, and reduce conflict and understand the resource dynamics (local conditions), which are underpinned by group cognitive abilities

2 Supplementary Note 2

2.1 Basic Statistics

Distributions and descriptive statistics for the data used are depicted below. The age distribution is consistent with that expected from a sample of undergraduate students. Individuals partici-

pating in the experiments are mainly majoring in social and behavioral sciences (social science and psychology).

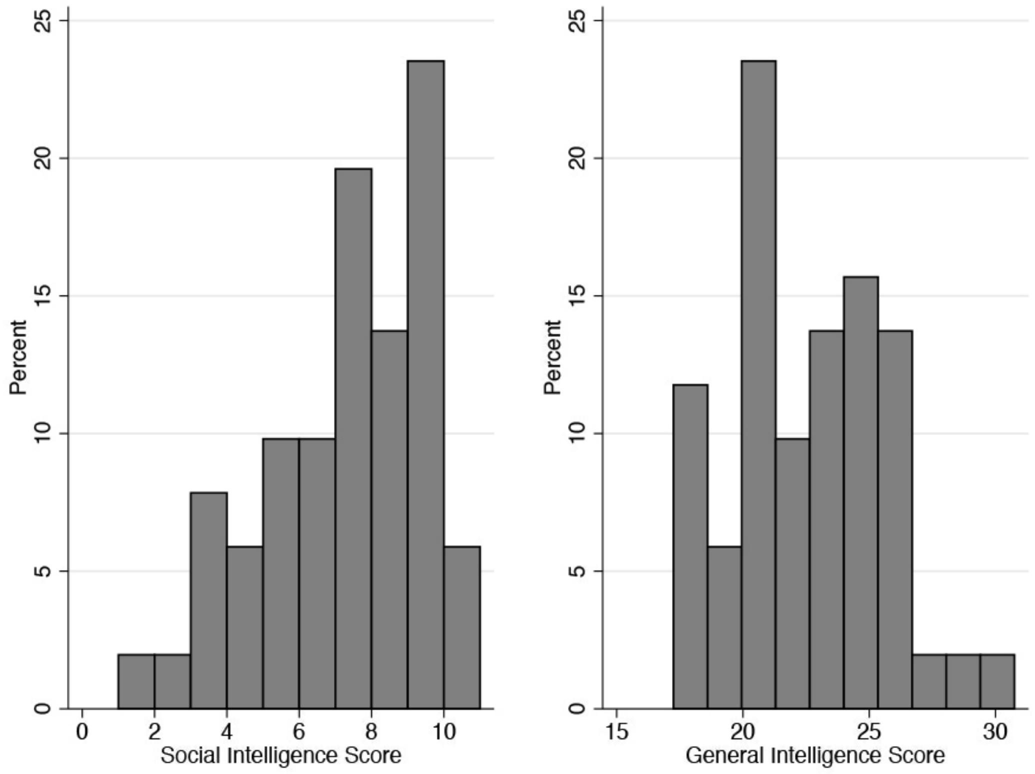

Figure 1: Distribution of social and general intelligence between groups. Social intelligence is derived from the SST test, general intelligence is scaled with respect to ACT scores - see methods in the main paper.

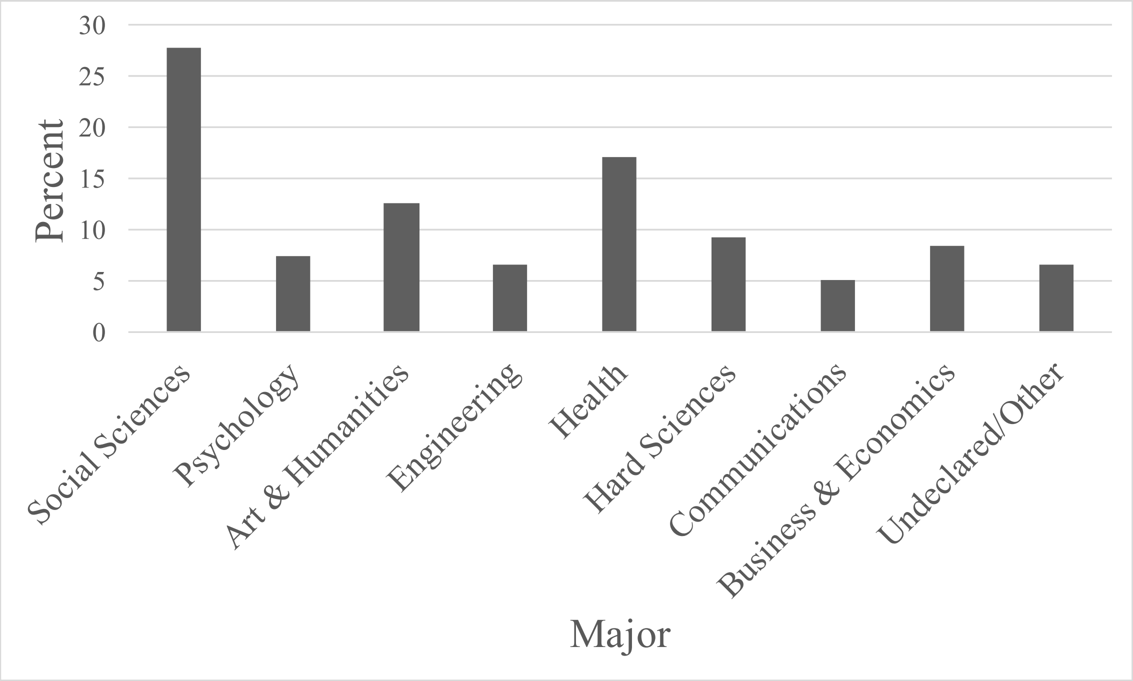

Figure 2: Declared majors of participating individuals

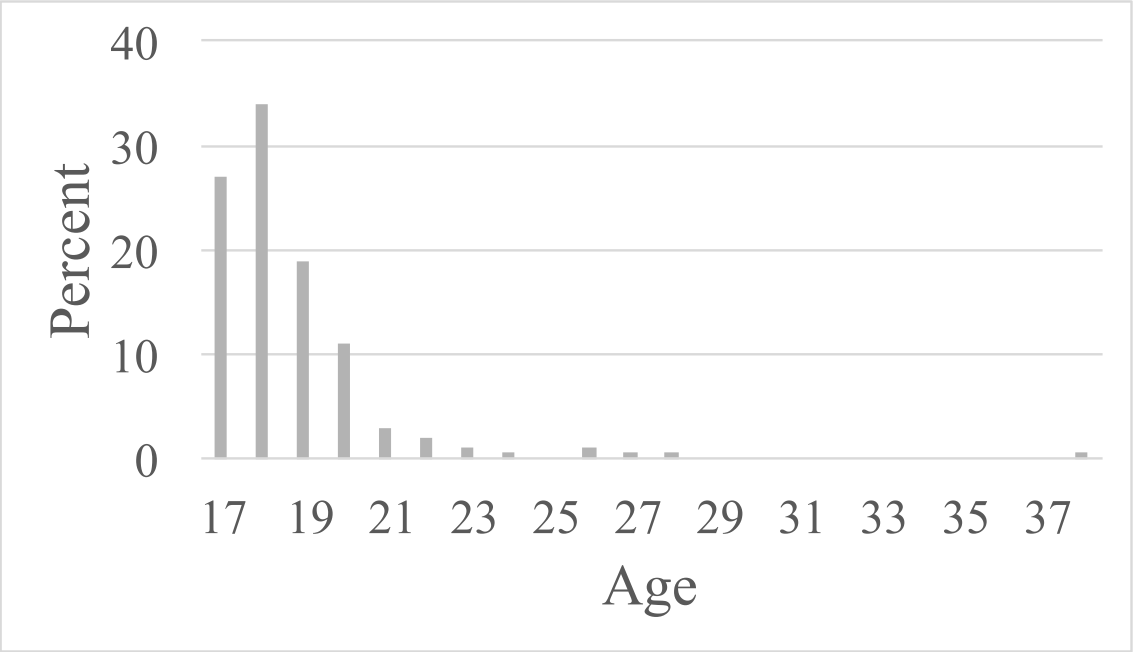

Figure 3: Age distribution of individuals participating in the experiments

Table 1: Descriptive Statistics for all variable used in the analysis

Variable	Mean	StDev	Median	$25^{th}_{\%}$	$75^{th}_{\%}$	Min	Max
General Intelligence	22.46	2.98	22.33	20.25	24.75	17.25	30.75
Social Intelligence	6.85	2.28	7.00	5.00	9.00	1.00	11.00
Trust	-0.71	1.93	-1.00	-2.00	1.00	-4.00	3.00
Avg Chat Volume	6.49	4.06	6.00	3.00	9.00	0.00	19.00
Ethnic Diversity	0.52	0.44	0.56	0.00	1.04	0.00	1.39
Religious Diversity	0.82	0.43	0.87	0.56	1.04	0.00	1.39
% Males	0.58	0.21	0.50	0.50	0.75	0.25	1.00

Table **1** portrays the descriptive statistics (mean, median, standard deviation, 25th and 75th percentiles, minimum and maximum) of all variables used in the analysis.

2.2 External Validity of our Sample

All experimental studies have issues with external validity, more so if performed in a western university with an undergraduate population. We compare here our average results with the wider U.S. population using data available from the Gallup Report for 2016 available at https://news.gallup.com/poll/200186/five-key-findings-religion.aspx. We find that our religious diversity is very much in line with the average religious diversity within the wider U.S. population. In fact, our average religious diversity metric 0.82 (see table 1 in the supplementary material) is very similar to the average religious diversity metrics of the U.S. when all Christian religions are considered together (0.84), albeit lower when different Christian religions are separated, as diversity then increases to 1.38 for the U.S. population.

Comparing the ethnic diversity of our sample size with the wider U.S. population we find that our sample composition is more homogeneous than the overall U.S. population, however, this also reflects the inbuilt biases that exist within the U.S. college population. In fact, while our ethnic diversity index (calculated as described in the main paper, method section) is = 0.53 on average, the U.S. population ethnic diversity index (dividing white and Hispanics) is = 1.1 – almost double. However, if we look at the % of individuals within each diversity index value,

while most groups are very homogeneous, over 30% of our groups reflect the overall U.S. average as shown below.

Ethnic Diversity Index	Percent
0	36.54
0.5623351	25.00
0.6931472	7.69
1.039721	28.85
1.386294	1.92

With respect to age, our sample is definitely skewed compared to the wider population given the age restriction and inbuilt bias that exist when sampling undergraduate students.

Unfortunately, no state level data are available for ToM. In a previous study, Freeman and colleagues [10] used agreeableness as a proxy for ToM. The metric we use in this study, based on a specific reasoning test, and the agreeableness metric Freeman et al. [10] are moderately correlated (see also [11] where a British version of the SST was correlated with agreeableness). However, as expected, our sample has a narrower spreading as well as higher g compared to population average with respect to the U.S.

3 Supplementary Method 1

The following sections report the model output that was used to calculate the marginal effects portrayed in Figures 2, 3 and 4 of the main paper, as well as additional analyses to assess whether the results may be a construct of the aggregation of individual cognitive abilities chosen. We first describes the simulations that we conducted to construct the response variables ΔT and $avgT$. Supplementary Method 2 describes the regression outputs portrayed in Figure 2 and 3 of the main paper. Supplementary Method 3 presents the results of regressions that examine the three-way interaction effects between g, ToM and ecological $change$. These models exclude the control variables because it is difficult to interpret three-way interaction effects, and fewer variables increases the robustness of interpretation. Note that we compare the three-way regressions with the two-way interaction regression that include control variables and observe consistent results. Finally, Supplementary Method 4 showcases results for the same models presented in Supplementary Method 2 but where g and ToM were aggregated differently using $avg(ToM)$ and $min(g)$.

3.1 Simulations

Two of our response variables depend on calculating the “optimal” number of potential tokens that a group could harvest, if that group followed the best cooperative protocol for harvesting tokens in the experimental environment. These response variables are ΔT and $avgT$.

ΔT computes the difference between the percentage of potential tokens collected in the first and second three rounds of each treatment. For instance, hypothetically, in the high-to-low treatment, if, on average, the maximum number of tokens that could be collected in rounds 1-3 was 100, and a group collected 50, the group would have a collected 50% of potential tokens. In the second three rounds with a lower growth rate, if the group collected 40/50 tokens, they would have collected 80% of tokens. In this example, $\Delta T = 50\% - 80\% = -30\%$. This is a

very low value not seen in our experiment and would indicate that the group, after the negative ecological change, more readily followed the best cooperative protocol (described below) for harvesting resources after the negative change to growth rate. In contrast, positive values of ΔT indicate that groups less readily followed the best cooperative protocol and either collapsed or severely depressed the resource after the ecological change.

$avgT$ is calculated by averaging the $\%$ of tokens collected individually for each round. More formally we calculate $avgT = \frac{\sum T_i}{MaxT}$ where $\sum T_i$ = sum of tokens collected by each individual of a specific group, and $MaxT$ = maximum number of potential token calculated via simulating an optimized token collection. The interpretation of this variable is intuitive. The higher the percentage, the more closely a group approximated the best cooperative protocol for harvesting tokens described by the simulation below.

To simulate the optimal harvest of tokens we created a 20X20 grid, of which 15$\%$ of cells are filled with tokens. Initially, simulated agents are placed in the middle row of the grid with equal distances between them. The setup of the simulation accurately resembles the start of an experimental round. Available actions for an agent in one step include move up, down, left, right, and collect a token. Simulated agents must move to the cell that contains a token and explicitly press the space bar to collect that token. Agents are allowed to have at most 10 actions per second. Agents are not allowed to overlap in a cell, which means an agent cannot move to a cell that is already occupied by another agent. The length of each round is 180 seconds. During a round, the complete information about the spatial position of tokens and actions of other members is available for an agent. Agents also have access to the number of tokens collected by other players while playing a round. An empty cell has a probability p_t of generating a new token. p_t is density-dependent on the number of adjacent cells with tokens. $p_t = p \times \frac{n_t}{N}$, where n_t is the number of adjacent cells containing a token, N is the number of adjacent cells ($N = 8$). The control probability p is set to 0.01 in the "high" configuration, and

0.05 in the “low” configuration. An empty cell surrounded with more tokens will have higher probability of generating a token compared to a cell surrounded by fewer tokens. A cell must have at least one adjacent cell filled with a token in order for a new token generation to appear. Thus, if agents collect all tokens in the grid, no additional token generation will occur.

The optimum level of collecting tokens depends on the initial starting conditions, the generating probabilities and the spatial variability of tokens. In theory, if we ignore the spatial variability, the optimal strategy is defined by a two step strategy: (1) Wait until the tokens grow to 50% density, which leads to the highest growth rate, and harvest at a rate that keeps the resource at a 50% density. (2) At the point where there is just enough time left to harvest every single token, harvest all of the tokens before time runs out. However, in our simulation, the number of tokens collected is also dependent on the spatial variability of the tokens. To calculate a distribution of the maximum number of tokens collected we performed 4,000 simulation rounds (2,000 for each type of configuration “high and low”) using the same strategy to maximize earnings as in previous research [12]. The strategy is described as follows.

At the beginning of a round, agents wait until the resource grows to 50% density. After that at each unit time (seconds) tokens with four tokens or more on the neighboring cells are collected, using a randomized non-sequential updating of the tokens. Tokens are collected but the harvesting rate keeps the resource at a 50% density. Our experimental settings does not allow more than 10 actions per second per agent, thus the maximum number of tokens collected per second is 40 for a four agent experiment. At the end of the experiment, tokens are collected such that not more than $40 * (secondsremaining)$ tokens remain on the screen. When clearing the grid, agents always move to the nearest token to collect. The following figure portrays the pseudocode for the simulation.

Because the initial token distribution and the control probability are small in our experiment, the number of tokens never reaches 50% density in the “low” configuration. Thus, the optimal

```
while TRUE do
    if The round is about to finish then
        Collect all remaining tokens in the grid
        Break
    end if
    if The number of tokens < 50% then
        Wait
    else
        while The number of tokens ≥ 50% do
            Collect tokens with four tokens or more on the neighboring cells
        end while
    end if
end while
```

Figure 4: Simulation pseudocode

strategy is simply to wait until the number of moves possible (10 moves per second) is just sufficient to collect all of the tokens on the grid. Figure 5 shows the histogram of the number of tokens collected in a round at the group level in the "low" configuration. We use the max of this distribution to approximate the optimal number of tokens harvested per group per round. Thus, the optimal number of collected tokens per group per round for the "low" configuration is 145, which means, at maximum, an agent collects 23.25 tokens. Figure 6 shows the result for the "high" configuration. On average, the optimal number of tokens collected at the group level in the "high" configuration is 220, or 55 tokens maximum per agent.

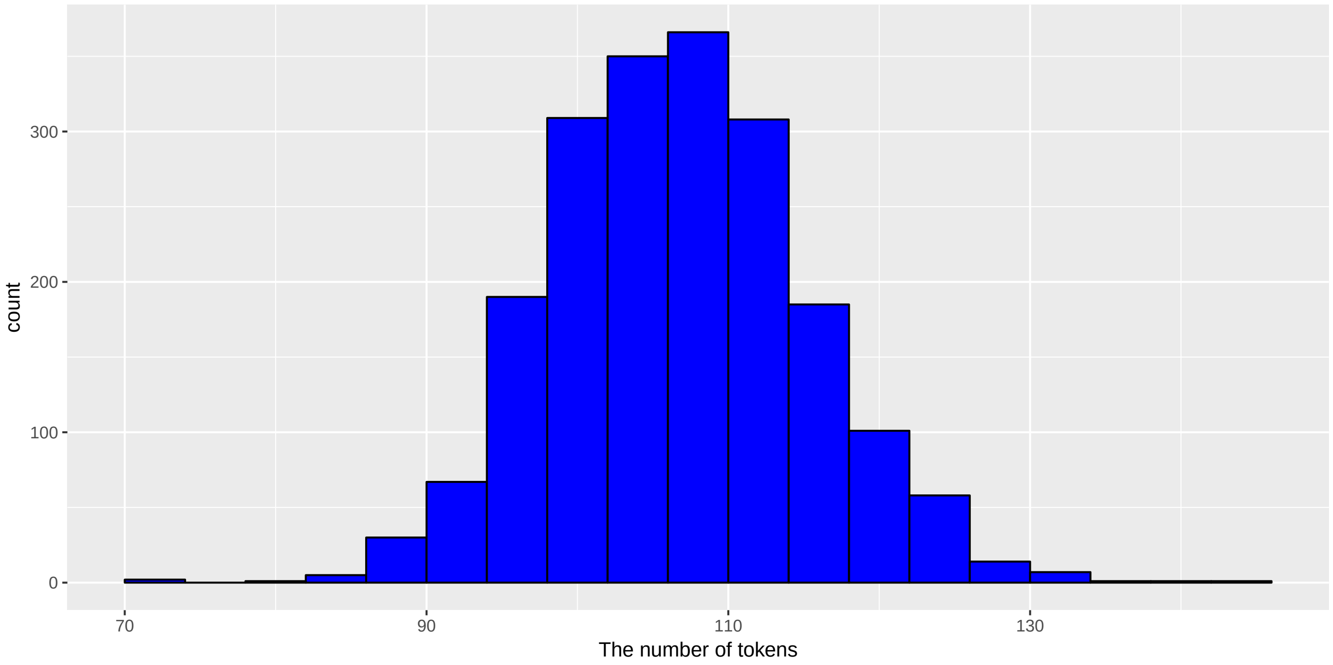

Figure 5: Simulation result for “low” configuration

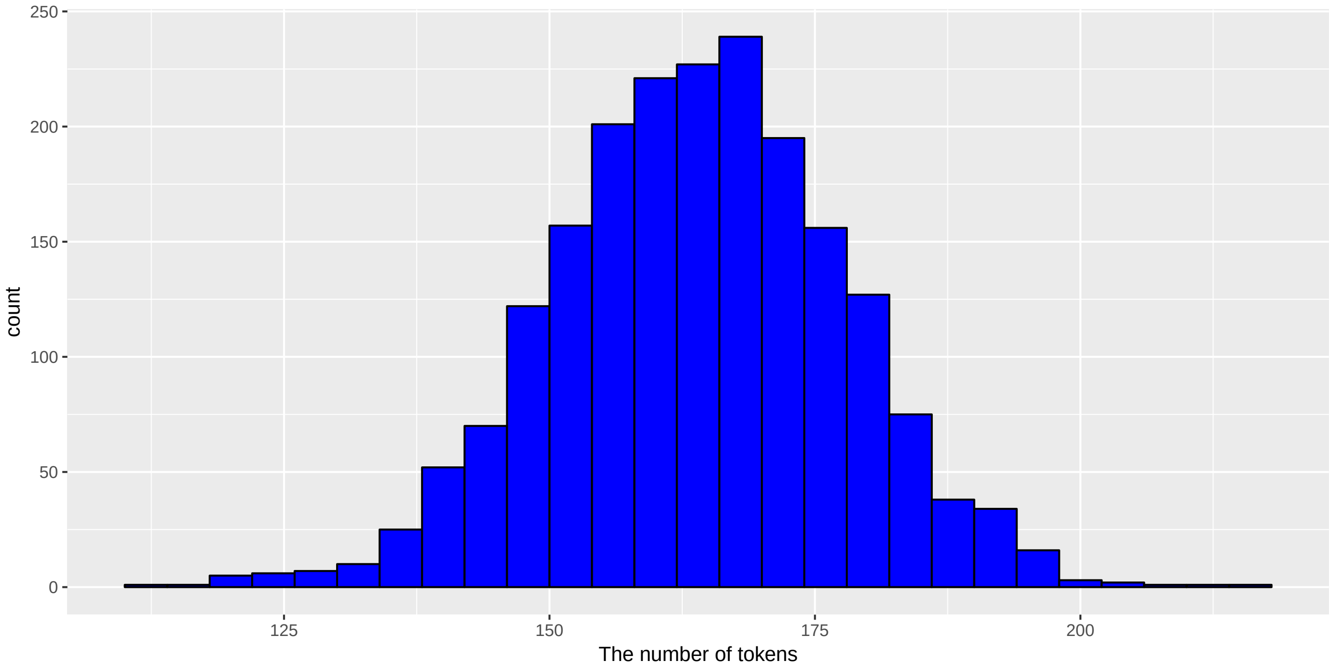

Figure 6: Simulation result for “high” configuration

4 Supplementary Method 2

4.1 Two way interaction effect regressions

Table 2 reports the statistical model output and factors affecting $Time$. With respect to $Time$, when conditions worsen - HL treatment - both ToM and g are always positive, meaning that taken singularly they increase the probability of quickly collapsing the resources and thus increase the time "spent staring at an empty screen". However, the interaction between g and ToM is negative, suggesting that the interplay between the different cognitive abilities reduces the probability of quickly collapsing resources. In the HL treatment, the interaction of g and ToM is always significant, as well as ToM, while g is significant in 4 out of 6 cases.

On the other hand, when conditions improve - LH treatment - g alone, on average, increases the probability of collapsing resources, while ToM alone decreases it. Once again, the interaction of g and ToM decreases the probability of quickly collapsing resources. However, notwithstanding the direction (positive/negative) and the average effect of both g and ToM, when conditions improve (LH models) cognitive abilities do not to have a statistically significant effect on the probability of quickly collapsing resources. When conditions improve, reciprocity–what happened in the previous round–is the main factor driving increases/decreases in the probability of quickly collapsing resources.

Table 2: General Linear Model: Individual cognitive abilities and controlling factors on $Time$

Predictors	HLt1	HLt2	HLt3	HLt4	HLt5	HLt6	LHt1	LHt2	LHt3	LHt4	LHt5	LHt6
$min(ToM)$	0.925*	0.925*	0.829	0.867*	1.210**	1.309***	0.213	0.254	0.258	0.208	0.181	0.224
	(0.483)	(0.484)	(0.540)	(0.514)	(0.476)	(0.501)	(0.591)	(0.584)	(0.598)	(0.611)	(0.620)	(0.612)
$avg(g)$	0.278*	0.278*	0.237	0.240	0.362**	0.404**	-0.078	-0.065	-0.062	-0.038	-0.040	-0.028
	(0.145)	(0.144)	(0.168)	(0.162)	(0.157)	(0.163)	(0.182)	(0.179)	(0.192)	(0.192)	(0.194)	(0.194)
$avg(g) * min(ToM)$	-0.058***	-0.058***	-0.054**	-0.056**	-0.072***	-0.077***	-0.008	-0.010	-0.010	-0.009	-0.007	-0.009
	(0.022)	(0.022)	(0.024)	(0.023)	(0.022)	(0.023)	(0.027)	(0.027)	(0.027)	(0.028)	(0.028)	(0.028)
Round 1 Baseline	0.000	0.000	0.000	0.000	0.000	0.000	0.000	0.000	0.000	0.000	0.000	0.000
	(.)	(.)	(.)	(.)	(.)	(.)	(.)	(.)	(.)	(.)	(.)	(.)
Round 2	-1.384***	-1.392***	-1.386***	-1.394***	-1.480***	-1.505***	-1.519***	-1.333***	-1.333***	-1.288***	-1.284***	-1.306***
	(0.434)	(0.442)	(0.442)	(0.444)	(0.419)	(0.418)	(0.461)	(0.494)	(0.494)	(0.498)	(0.495)	(0.494)
Round 3	-1.715***	-1.726***	-1.719***	-1.728***	-1.836***	-1.868***	-1.230***	-1.060**	-1.060**	-1.018**	-1.016**	-1.036**
	(0.460)	(0.496)	(0.494)	(0.493)	(0.461)	(0.456)	(0.413)	(0.441)	(0.441)	(0.446)	(0.452)	(0.458)
Round 4	-1.558***	-1.569***	-1.562***	-1.571***	-1.676***	-1.709***	-1.963***	-1.859***	-1.859***	-1.837***	-1.838***	-1.852***
	(0.457)	(0.452)	(0.452)	(0.458)	(0.443)	(0.422)	(0.440)	(0.452)	(0.452)	(0.457)	(0.453)	(0.456)
Round 5	-1.891***	-1.902***	-1.895***	-1.904***	-2.019***	-2.053***	-2.293***	-2.183***	-2.182***	-2.159***	-2.157***	-2.173***
	(0.388)	(0.408)	(0.407)	(0.409)	(0.388)	(0.381)	(0.448)	(0.461)	(0.461)	(0.470)	(0.469)	(0.473)
Round 6	-2.276***	-2.286***	-2.280***	-2.288***	-2.405***	-2.435***	-2.415***	-2.355***	-2.355***	-2.345***	-2.343***	-2.352***
	(0.417)	(0.451)	(0.450)	(0.453)	(0.452)	(0.451)	(0.493)	(0.493)	(0.493)	(0.502)	(0.504)	(0.509)
Chat		0.002	0.001	0.002	0.015	0.022		-0.045	-0.045	-0.058	-0.059	-0.054
		(0.043)	(0.042)	(0.042)	(0.039)	(0.038)		(0.039)	(0.039)	(0.040)	(0.040)	(0.045)
Trust			0.046	0.049	0.129	0.113			-0.004	-0.017	-0.005	0.006
			(0.092)	(0.090)	(0.092)	(0.089)			(0.085)	(0.085)	(0.088)	(0.097)
Religious Diversity				-0.247	-1.123***	-1.437**				0.507	0.341	0.329
				(0.334)	(0.421)	(0.576)				(0.391)	(0.506)	(0.514)
Ethnic Diversity					1.414***	1.608***					0.296	0.274
					(0.430)	(0.538)					(0.450)	(0.443)
Gender						0.670						0.267
						(0.772)						(0.930)
Constant	-4.126	-4.143	-3.185	-3.018	-5.683*	-6.742*	1.445	1.319	1.255	0.532	0.490	0.092
	(3.147)	(3.164)	(3.667)	(3.551)	(3.374)	(3.602)	(3.963)	(3.886)	(4.213)	(4.210)	(4.233)	(4.305)
N	162	162	162	162	162	162	150	150	150	150	150	150
AIC	104.320	106.318	108.242	110.096	109.395	111.187	127.728	129.199	131.199	132.579	134.396	136.359
D	0.215	0.217	0.218	0.218	0.202	0.202	0.385	0.384	0.387	0.385	0.387	0.390

Note: Robust standard errors reported in parenthesis * = significant at the 90% level, ** = significant at the 95% level, and *** = significant at the 99% level. Round 1 = baseline to which other rounds are compared to. HLr1 to HLr6 represent 6 regression equations estimated hierarchically, adding one new predictor for each equation, for the condition in which participants go from high to low resource growth rates. LH r1 to LHr6 represent the condition in which participants go from low to high resource growth rates.

Table 3 portrays the statistical model output and factors affecting ΔT. With respect to ΔT, we observe the same results as with respect to $Time$. Groups with high ToM or g alone are more likely to increase harvest pressure after a change in resource regrowth and thus overharvest (i.e., collect tokens faster than the regrowth rate). Cognitive abilities are statistically significant in the case of pejorative conditions - HL treatment - and in case of improved conditions -LH treatment -. In all cases, ToM and g alone increase pressure on natural resources after conditions either deteriorate or improve. That is, both g and ToM diplay positive signs (increase in g or ToM increases ΔT and hence increases pressure on resources after a change). On the other hand, the interaction between g and ToM reduces pressure on resources (negative sign).

Groups with high ToM or g are more prone to increase harvest pressure after a change in resource regrowth, hence reducing the ability of groups to harvest resources sustainably, especially in case of a pejorative change (see also Figures 2 and 3 in the main paper and Tables 2 and 3).

Table 3: OLS Regression: Individual cognitive abilities and controlling factor on ΔT

Predictors	HLr1	HLr2	HLr3	HLr4	HLr5	HLr6	LHr1	LHr2	LHr3	LHr4	LHr5	LHr6
$min(ToM)$	0.065***	0.065***	0.096***	0.096***	0.096***	0.101***	0.074***	0.068**	0.056*	0.086***	0.089***	0.136***
	(0.015)	(0.015)	(0.012)	(0.011)	(0.012)	(0.012)	(0.026)	(0.028)	(0.028)	(0.032)	(0.032)	(0.034)
$avg(g)$	0.023***	0.023***	0.036***	0.037***	0.037***	0.038***	0.044***	0.042***	0.033***	0.034***	0.031***	0.046***
	(0.005)	(0.005)	(0.004)	(0.004)	(0.004)	(0.004)	(0.007)	(0.008)	(0.009)	(0.010)	(0.009)	(0.009)
$avg(g) * min(ToM)$	-0.004***	-0.004***	-0.005***	-0.005***	-0.005***	-0.005***	-0.004***	-0.004***	-0.003**	-0.004***	-0.004***	-0.007***
	(0.001)	(0.001)	(0.001)	(0.000)	(0.001)	(0.001)	(0.001)	(0.001)	(0.001)	(0.001)	(0.001)	(0.002)
Chat Volume		-0.000	0.004***	0.003***	0.003***	0.004***		-0.003	-0.003	0.001	0.000	0.002
		(0.001)	(0.001)	(0.001)	(0.001)	(0.001)		(0.006)	(0.006)	(0.005)	(0.004)	(0.004)
Trust			-0.015***	-0.014***	-0.014***	-0.014***			0.012**	0.015***	0.012**	0.020***
			(0.002)	(0.002)	(0.002)	(0.002)			(0.005)	(0.005)	(0.006)	(0.007)
Religious Diversity				0.015*	0.012	0.028**				-0.120***	-0.063	-0.059
				(0.009)	(0.011)	(0.013)				(0.040)	(0.050)	(0.043)
Ethnic Diversity					0.005	-0.005					-0.095***	-0.120***
					(0.012)	(0.014)					(0.031)	(0.034)
Gender						-0.047**						0.273***
						(0.023)						(0.098)
Constant	-0.238**	-0.238**	-0.546***	-0.575***	-0.579***	-0.593***	-0.884***	-0.832***	-0.643***	-0.588***	-0.519**	-0.971***
	(0.109)	(0.109)	(0.090)	(0.086)	(0.089)	(0.094)	(0.156)	(0.181)	(0.198)	(0.218)	(0.203)	(0.210)
N	27	27	27	27	27	27	25	25	25	25	25	25
AIC	-508.815	-506.829	-544.754	-546.404	-544.602	-548.054	-159.525	-157.666	-158.390	-171.440	-177.346	-195.748
R^2	0.439	0.440	0.562	0.572	0.572	0.586	0.122	0.123	0.139	0.221	0.261	0.355

Note: Standardized coefficients reported. Robust standard errors reported in parenthesis * = significant at the 90% level, ** = significant at the 95% level, and *** = significant at the 99% level. ΔT represents the change in tokens collected between rounds 1-3 and rounds 4-6 (i.e. before and after the ecological change). HLr1 to HLr6 represent 6 regression equations estimated hierarchically, adding one new predictor for each equation, for the condition in which participants go from high to low resource growth rates. LH r1 to LHr6 represent the condition in which participants go from low to high resource growth rates.

Figure 7 represent marginal effects of ToM and g on $Time$ and ΔT for models HLt1 and LHt1 in Table 2 and models HLr1 and LHr1 in Table 3 for selected values of g and ToM.

When environmental condition worsen (HL models) groups with high levels of both ToM and g perform better than groups with either high ToM or high g (as represented by $ToM = 11andg = 31$. The main difference between HL and LH models is clearly shown in Figure 7 by the difference between the reduction in $Time$ that is independent of the level of ToM in LH models (panel Time LH), and seem to have a ceiling effect on g. $Time$ and ΔT as stated in Supplementary Note 2 are not related in LH models, that is, more pressure on resources does not provoke an increase in speed of resource depletion. Finally, it is important to note that Figure 7 represent the same results portrayed in Figures 2 and 3 in the main text. The difference being that in the main text both ToM and g are the axis while the average $Time$ or ΔT is represented by the color.

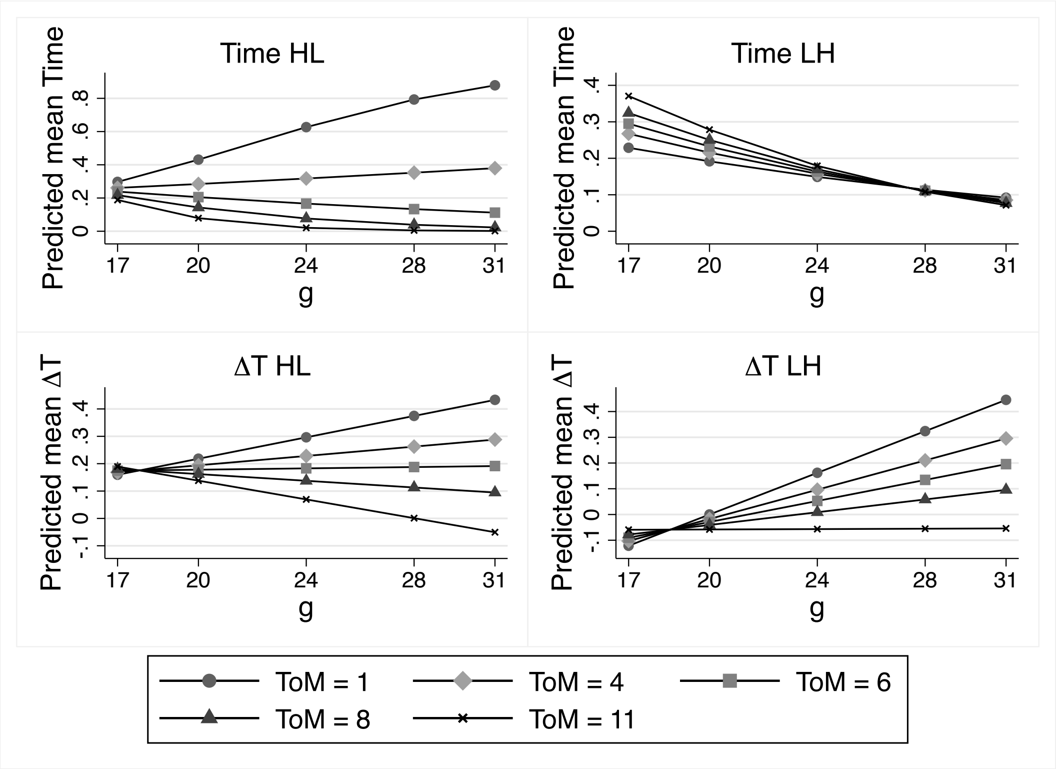

Figure 7: Marginal effects for HLt1, LHt1 models in Table 2 and HLr1 and LHr1 models in Table 3

5 Supplementary Method 3

5.1 Three way interaction effect regressions

Table 4 portrays the results of regressions that include three way interactions between cognitive abilities and ecological changes on $Time$. Here we first analyze the effect of g, ToM and $change$ on $Time$ without interaction (models HLt1 and LHt1, Table 4). $change$ is a dummy variable that assumes value 0 in the first three rounds pre growth rate change, and value 1 in the second three rounds post growth rate change. We then analyze the interaction between g and ToM for the first three rounds (model HLtB and HLtA, Table 4) and for the second three rounds (models HLtA and LHtA, Table 4). Finally we analyze the three way interaction betweeen g, ToM and $change$ (models HLt4 and LHt4, Table 4). It is important to remember here, that the lower $Time$ is, the longer groups are able to harvest. Hence high values of $Time$ represent faster resource collapse.

In Table 4, both ToM and g have a negative and independent effect in the HL treatment. However, when we add an interaction term for g and ToM, the interaction is significant and negative, while g and ToM have a positive effect on $Time$; that is, on their own, g and ToM contribute to a faster resource collapse, but together they slow the resource collapse, or avoid it ($Time = 0$). This result is consistent with Figure 2 in the main text. In LH models, the interaction term is negative and significant only in the last 3 rounds; after the change in re-growth rate (model LHr A, Table 4. Overall, when conditions improve (see LH models Table 4) reinforces the results presented in Table 2: cognitive abilities are less likely to have a significant effect on group ability to manage resources sustainably. Generally speaking, groups are more likely to perform worse after $change$ in HL models ($change > 0$ in model HLr4, Table 4).

The results portrayed in Table 4 are also illustrated in Figure 8. Figure 8 showcases the marginal effects of g and ToM before and after ecological changes. HLt1-b and LHt1-b repre-

sent the marginal effects of cognitive abilities when $change = 0$ (rounds 1-3) for models HLt1 and LHt1 in Table 4, and HLt1-a as well as LHt1-a represent the marginal effects of cognitive abilities when $change = 1$ (rounds 4-6) for the same models (HLt1 andf LHt1 in Table 4). Panel HLt B and LHtB represent marginal effects of g and ToM for rounds 1-3 (before change) and reported in model HLt B and LHt B in Table 4. Finally, panels HLt4-b and LHt4-b represent the marginal effects of cognitive abilities when $change = 0$ (rounds 1-3) for models HLt4 and LHt4 in Table 4, and HLt4-a as well as LHt4-a represent the marginal effects of cognitive abilities when $change = 1$ (rounds 4-6) for the same models (HLt4 andf LHt4 in Table 4).

Here we can see how groups are able to manage resources sustainably (or not) ($Time$) for different levels of g and ToM. Similar to the results portrayed in Figure 2 in the main paper and Table 2 above, groups with high g but low ToM (Figure 8 lower right corner of plots) or high ToM but low g (Figure 8 upper left corner of plots) perform worse than groups with high g and high ToM (Figure 8 upper right corner of plots). When g increases, at low levels of ToM, a group is more likely to collapse the resource (i.e., $Time$ is higher). Generally speaking, before and after the change, in both HL and LH models, groups with high g and high ToM are more able to manage resources sustainably (dark blue area in the upper right corner).

Table 4: Effect of cognitive abilities and perturbation on $Time$

	HLt1	HLtB	HLtA	HLt4	LHt1	LHtB	LHtA	LHt4
$min(ToM)$	-0.301***	1.361*	0.246	1.361*	0.046	-0.216	1.958**	-0.216
	(0.062)	(0.720)	(0.498)	(0.718)	(0.063)	(0.683)	(0.995)	(0.680)
$avg(g)$	-0.101*	0.459**	-0.004	0.459**	-0.134**	-0.175	0.332	-0.175
	(0.052)	(0.208)	(0.152)	(0.207)	(0.061)	(0.228)	(0.251)	(0.228)
change	-2.276***				-2.414***			
	(0.453)				(0.496)			
Round 1	0.000	0.000		0.000	0.000	0.000		0.000
	(.)	(.)		(.)	(.)	(.)		(.)
Round 2	-1.369***	-1.499***		-1.499***	-1.518***	-1.506***		-1.506***
	(0.440)	(0.464)		(0.462)	(0.460)	(0.449)		(0.447)
Round 3	-1.706***	-1.849***		-1.849***	-1.229***	-1.219***		-1.219***
	(0.453)	(0.495)		(0.493)	(0.413)	(0.409)		(0.408)
Round 4	0.729		0.000	0.690*	0.452		0.000	0.480
	(0.457)		(.)	(0.374)	(0.559)		(.)	(0.516)
Round 5	0.391		-0.319	0.372	0.122		-0.351	0.129
	(0.365)		(0.374)	(0.318)	(0.566)		(0.487)	(0.503)
Round 6	0.000		-0.690*	0.000	0.000		-0.480	0.000
	(.)		(0.375)	(.)	(.)		(0.518)	(.)
$min(ToM) * avg(g)$		-0.085***	-0.018	-0.085***		0.015	-0.099**	0.015
		(0.032)	(0.022)	(0.032)		(0.032)	(0.047)	(0.031)
B_{change}				0.000				0.000
				(.)				(.)
A_{change}				5.666				-11.658
				(5.675)				(7.278)
$B_{change} * min(ToM)$				0.000				0.000
				(.)				(.)
$A_{change} * min(ToM)$				-1.114				2.174*
				(0.873)				(1.202)
$B_{change} * avg(g)$				0.000				0.000
				(.)				(.)
$A_{change} * avg(g)$				-0.463*				0.507
				(0.257)				(0.338)
$B_{change} * min(ToM) * avg(g)$				0.000				0.000
				(.)				(.)
$A_{change} * min(ToM) * avg(g)$				0.067*				-0.114**
				(0.039)				(0.056)
Constant	3.702***	-7.112	-0.756	-7.112	2.627**	3.093	-8.084	3.093
	(1.140)	(4.663)	(3.309)	(4.649)	(1.325)	(4.931)	(5.461)	(4.915)
N	162.000	81.000	81.000	162.000	150.000	75.000	75.000	150.000
AIC	103.783	60.475	48.042	108.517	125.762	81.563	48.384	129.947
D	0.224	0.297	0.118	0.208	0.383	0.525	0.208	0.366

Note: Robust standard errors reported in parenthesis * = significant at the 90% level, ** = significant at the 95% level, and *** = significant at the 99% level. Round 1 = baseline to which other rounds are compared to. $change$ = dummy variable representing before B_{change} and after A_{change} the ecological change. B_{change} is used as baseline. HL represent 4 regression equations estimated assessing the relationship between perturbation, g and ToM and their effect on $Time$ in case of pejorative conditions (high to low resource growth rate). LH represent the condition in which participants go from low to high resource growth rates. HL or LH B = takes into only account rounds before the shock (round 1 used as baseline). HL or LH A = takes into account only rounds after the change (round 4 used as baseline)

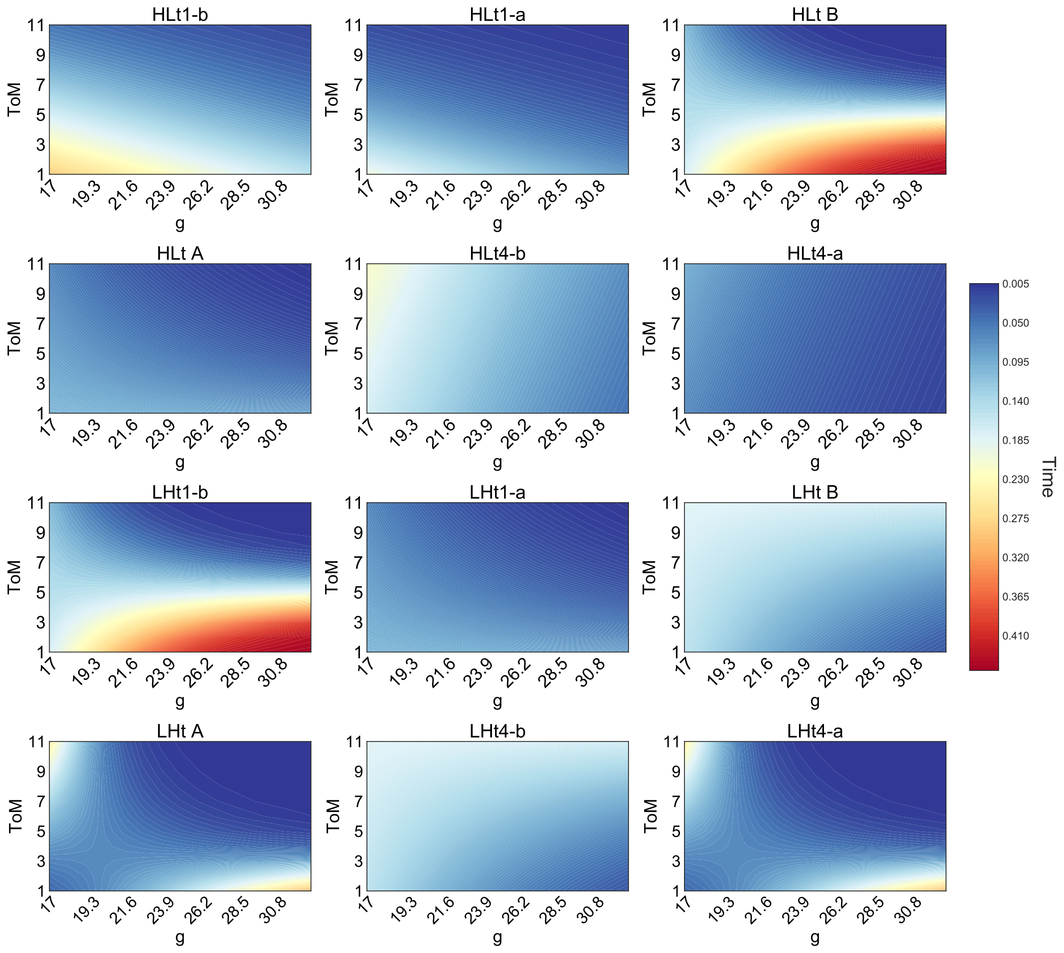

Figure 8: Effects of g and ToM and $change\ Time$. Blue color = group harvested for more time (i.e. did not or collapsed the resource later). HL = high-to-low resource growth treatment, LH = low-to-high resource growth treatment. -b = marginal effect when $change = 0$ (before change); -a = marginal effects when $change = 1$ (after change). B = marginal effects taking only into account rounds 1-3 (before change). A = marginal effects calculated taking only into account rounds 4-6 (after change). *The different sub-figures represent the marginal effects of g and ToM for the different models portrayed in SI Table 4*

.

In Table 5 we investigate the effects of g, ToM and ecological $change$ on $avgT$. In order to run a three way interaction including tokens collected before and after the resource change, we are unable to employ ΔT as it is calculated as the difference between round 1-3 and 4-6. A three way interaction variable is impossible for this response variable. However, we can assess the effect of cognitive abilities on the average tokens collected as a % of the theoretically maximum number of tokens that could have been collected per round as the dependent variable ($avgT$). This allows us to assess how cognitive abilities affect overall $avgT$ before and after the ecological change.

To further investigate the effects of g, ToM and their interaction on group ability to manage and harvest resources, we then analyze the interaction between g and ToM for the first three rounds (model HLtB and HLtA) and for the second three rounds (models HLtA and LHtA). Finally we analyz the three way interaction betweeen g, ToM and $change$ (models HLt4 and LHt4).

In this last analysis, based on the results reported in Table 5, there is, again, a clear difference between improving and worsening conditions (HL and LH models). In HL models (worsening conditions) an increase in g increases $avgT$ when not interacted and when only taking into account rounds 4-6 (after change), see models HLr1 and HLrA in Table 5. However, an increase in g reduces $avgT$ when interacted with both, ToM and $change$ and when taking into account only rounds 1-3 (before change), see models HLrB and HLr4 in Table 5. On the other hand, in LH models, an increase in g always increases $avgT$: see models LHr1 LhrB LHrA and LHr4 in Table 5. In HL models an increase in ToM increases token harvested only when not interacted, see model HLr1, Table 5. In all other models an increase in ToM reduces $avgT$: see models HLrB, HLrA, HLr4, Table 5. In LH models, an increase in ToM reduces $avgT$ when not interacted, see model LHr1, Table 5; however an increase in ToM increases $avgT$ in all other LH models: see LHrB, LHrA, LHr4 in Table 5.

In HL models g is significant only when not interacted (model HLr1, Table 5). However, in LH models g is always significant. On the other hand, in HL models, ToM is significant when not interacted (model HLr1, Table 5), before the change (model HLrB, Table 5) and when interacted with both, g and $change$ (model HLr4, Table 5). In LH models, ToM is significant only in rounds 4-6 (after the change, model LHrA,Table 5).

The interaction term $g * ToM$ is positively associated with $avgT$: an increase in $g * ToM$ increases $avgT$ in all HL models (see models HLrB, HLrA and HLr4, Table 5), albeit being significant only when rounds 1-3 are taken into account, or when also interacted with $change$ (models HLrB and HLr4, Table 5). On the other hand, in LH models the interaction term is negatively related with $avgT$: an increase in $g * ToM$ reduces $avgT$: see models LHrB, LHrA and LHr4, Table 5. Further, $g * ToM$ is significant only after the change (model LHrA, Table 5).

These results are also shown graphically in Figure 9. In Figure 9, panels HLr1-b and LHr1-b represent the marginal effects of cognitive abilities when $change = 0$ (rounds 1-3) for models HLr1 and LHr1 in Table 5, and HLr1-a as well as LHr1-a represent the marginal effects of cognitive abilities when $change = 1$ (rounds 4-6) for the same models (HLr1 andf LHr1 in Table 5). Panel HLrB and LHrB represent marginal effects of g and ToM for rounds 1-3 (before change) and reported in model HLrB and LHrB in Table 5. Panels HLrA and LHrA represent marginal effects of g and ToM for rounds 4-6 (after change) and reported models HLrA and LHrA in Table 5. Finally, panels HLr4-b and LHr4-b represent the marginal effects of cognitive abilities when $change = 0$ (rounds 1-3) for models HLr4 and LHr4 in Table 5, and HLr4-a as well as LHr4-a represent the marginal effects of cognitive abilities when $change = 1$ (rounds 4-6) for the same models (HLr4 and LHr4 in Table 5). Figure 9, once again, reiterates the importance of both high g and ToM.

Fig.9 portrays the importance of both, high g and high ToM. In fact the figure portrays

how groups with higher competency in both cognitive abilities are able to harvest closer to the optimal level (interacted or not), as indicated by the dark blue color on the top right corner of panels HLrB, HLrA and HLr4 of Fig.9. Further, while HLr1-b, HLrB and HLr4-b all indicate marginal effects of g and ToM before thelogical change, Fig.9 panels HLr1-a, HLrA and HLr4-a indicate the marginal effects of g and ToM after the ecological change. In HLr1-b and HLr1-a there is no interaction effect between g and ToM (see also Table 4) but marginal effects are calculated respectively before and after the change. In panel HLrA and HLrB the regression includes the interaction effect between g and ToM but the model is run only for rounds 1-3 (model HLrB, Fig.9) or rounds 4-6 (model HLrA, Fig.9). Finally, panel HLr4 showcases the three way interaction effect between g, ToM and ecological $change$ as analyzed in model HLr4, Table 4. Panels HLr4-b and HLr4-a indicate marginal effects of g and ToM before and after the ecological change respectively. In all three panels, when both g and ToM are high, groups harvest a greater percentage of potential tokens because they do not collapse the resource base as readily as groups only high in g or ToM. In contrast, prior to the resource change in the LH treatment, groups with high g do better than groups with high g and high ToM or just as well. In other words, when conditions improve, groups with high g are better able to take advantage of the improved conditions and hence are able closer to the optimal level (the dark blue is in the lower right-hand corner of all effect plots labeled LH in Fig.9).

In sum, it is clearly noticeable that in the case of a negative change (HL treatment), $avgT$ is higher when both g and ToM are high. However, in the case of improving conditions, g is the cognitive ability that more clearly increases overall $avgT$ (see also main text).

Table 5: Effect of cognitive abilities and perturbation on $avgT$

	HLr1	HLrB	HLrA	HLr4	LHr1	LHrB	LHrA	LHr4
min(ToM)	0.012***	-0.064**	0.001	-0.064**	-0.002	0.028	0.103**	0.028
	(0.002)	(0.025)	(0.027)	(0.025)	(0.004)	(0.029)	(0.045)	(0.029)
avg(g)	0.016***	-0.009	0.014	-0.009	0.016***	0.015*	0.060***	0.015*
	(0.002)	(0.008)	(0.010)	(0.008)	(0.003)	(0.008)	(0.010)	(0.008)
change	0.152***				-0.005			
	(0.011)				(0.020)			
$min(ToM) * avg(g)$		0.004***	0.000	0.004***		-0.001	-0.005**	-0.001
		(0.001)	(0.001)	(0.001)		(0.001)	(0.002)	(0.001)
B_{change}				0.000				0.000
				(.)				(.)
A_{change}				-0.238				-0.884***
				(0.277)				(0.287)
$B_{change} * min(ToM)$				0.000				0.000
				(.)				(.)
$A_{change} * min(ToM)$				0.065*				0.074
				(0.036)				(0.053)
$B_{change} * avg(g)$				0.000				0.000
				(.)				(.)
$A_{change} * avg(g)$				0.023*				0.044***
				(0.013)				(0.013)
$B_{change} * min(ToM) * avg(g)$				0.000				0.000
				(.)				(.)
$A_{change} * min(ToM) * avg(g)$				-0.004**				-0.004*
				(0.002)				(0.002)
Constant	0.049	0.545***	0.307	0.545***	0.260***	0.227	-0.657***	0.227
	(0.039)	(0.175)	(0.214)	(0.175)	(0.070)	(0.166)	(0.234)	(0.166)
N	162.000	81.000	81.000	162.000	150.000	75.000	75.000	150.000
AIC	-408.450	-209.946	-213.722	-423.624	-203.736	-113.716	-93.575	-205.943
R^2	0.698	0.658	0.420	0.738	0.099	0.044	0.227	0.158

Note: Robust standard errors reported in parenthesis * = significant at the 90% level, ** = significant at the 95% level, and *** = significant at the 99% level. ΔT represents the change in tokens collected between the rounds 1-3 and the rounds 4-6. $change$ = dummy variable representing before B_{change} and after A_{change} the ecological change. HL represents 4 regression equations estimated assessing the relationship between perturbation, g and ToM and their effect on ΔT in case of pejorative conditions (high to low resource growth rate). LH represent the condition in which participants go from low to high resource growth rates. B = takes into only account rounds before the shock (round 1 used as baseline). A = takes into account only rounds after the change (round 4 used as baseline)

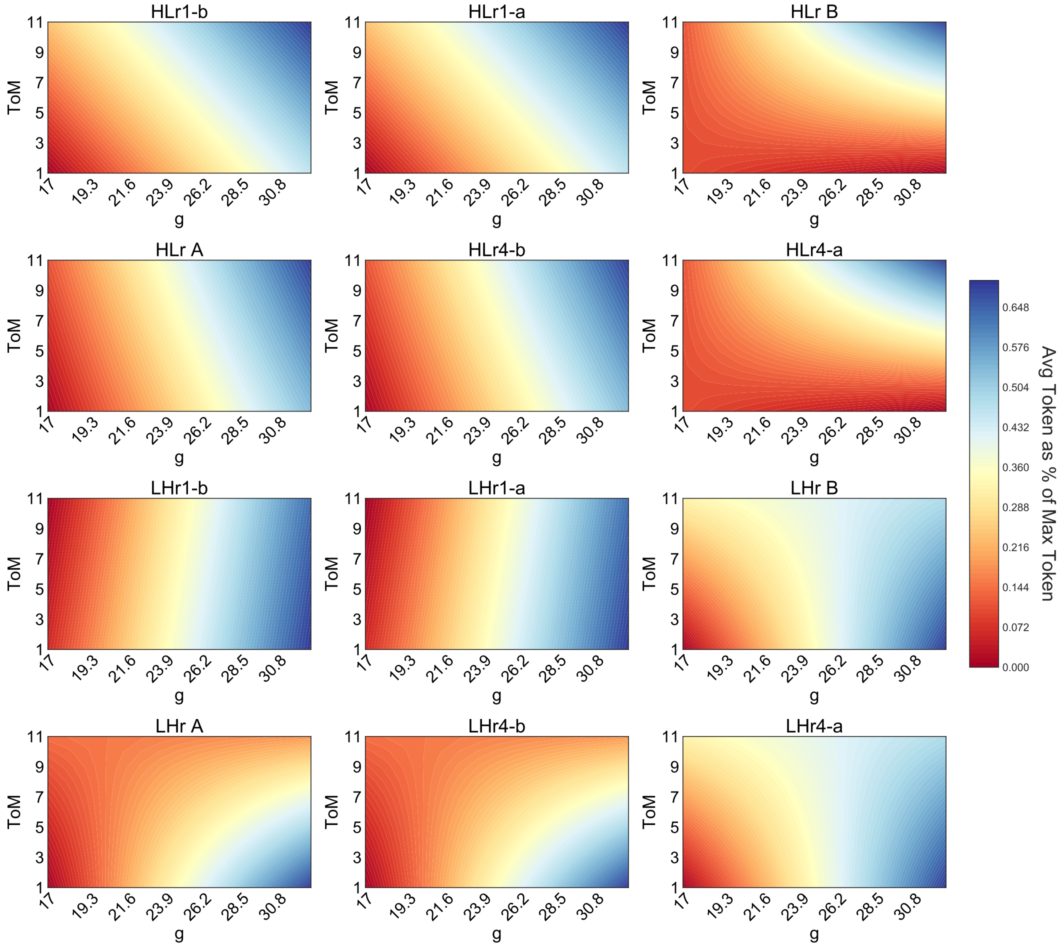

Figure 9: Effects of g and ToM and $change$ average token collected as % of maximum token available. Blue color = group harvested less. HL = high-to-low resource growth treatment, LH = low-to-high resource growth treatment. -b = marginal effect when $change = 0$ (before change); -a = marginal effects when $change = 1$ (after change). B = marginal effects taking only into account rounds 1-3 (before change). A = marginal effects calculated taking only into account rounds 4-6 (after change). *The different sub-figures represent the marginal effects of* g *and* ToM *for the different models portrayed in SI Table 5.*

6 Supplementary Method 4

6.1 Additional Models, changing aggregation of g and ToM

Although we assess avg g and min ToM as the main indicators of group cognitive abilities, in the following table we assess the same statistical models presented in Tables 2 and 3 using either $min(g)$ or $avg(ToM)$ on both $Time$ and ΔT. The results are consistent with those presented in the main paper.

Table 6: OLS Regression: Individual cognitive abilities and controlling factor on ΔT using $avg(ToM)$ in groups

Dep. Var.	HLsr1	HLsr2	HLsr3	HLsr4	HLsr5	HLsr6	LHsr1	LHsr2	LHsr3	LHsr4	LHsr5	LHsr6
$avg(ToM)$	0.071*	0.076**	0.132***	0.146***	0.155***	0.145***	0.075*	0.067	0.050	0.039	0.044	0.097**
	(0.036)	(0.038)	(0.036)	(0.035)	(0.037)	(0.038)	(0.045)	(0.045)	(0.047)	(0.052)	(0.052)	(0.048)
$avg(g)$	0.028*	0.027*	0.058***	0.065***	0.068***	0.063***	0.053***	0.048***	0.033*	0.016	0.016	0.040**
	(0.015)	(0.015)	(0.014)	(0.014)	(0.016)	(0.016)	(0.017)	(0.017)	(0.019)	(0.022)	(0.022)	(0.019)
$avg(g) * avg(ToM)$	-0.004**	-0.004**	-0.006***	-0.007***	-0.007***	-0.007***	-0.004**	-0.004*	-0.003	-0.001	-0.002	-0.005**
	(0.002)	(0.002)	(0.001)	(0.001)	(0.002)	(0.002)	(0.002)	(0.002)	(0.002)	(0.002)	(0.002)	(0.002)
$\Delta Chat$		0.004**	0.006***	0.006***	0.006***	0.007***		-0.006	-0.007	-0.007	-0.007	-0.005
		(0.002)	(0.001)	(0.001)	(0.002)	(0.002)		(0.006)	(0.006)	(0.005)	(0.005)	(0.005)
Trust			-0.016***	-0.016***	-0.017***	-0.017***			0.013**	0.019***	0.015**	0.022***
			(0.002)	(0.002)	(0.003)	(0.003)			(0.005)	(0.006)	(0.007)	(0.008)
Religious Diversity				0.016**	0.027*	0.037**				-0.122***	-0.070	-0.058
				(0.008)	(0.014)	(0.016)				(0.041)	(0.053)	(0.045)
Ethnic Diversity					-0.018	-0.025					-0.085***	-0.109***
					(0.016)	(0.016)					(0.032)	(0.034)
Gender						-0.032						0.245**
						(0.026)						(0.098)
Constant	-0.389	-0.427	-1.089***	-1.250***	-1.338***	-1.225***	-1.021***	-0.939**	-0.668	-0.289	-0.260	-0.862**
	(0.353)	(0.368)	(0.339)	(0.334)	(0.359)	(0.369)	(0.362)	(0.365)	(0.410)	(0.460)	(0.468)	(0.407)
N	27	27	27	27	27	27	25	25	25	25	25	25
AIC	-447.568	-449.216	-481.275	-481.767	-481.228	-480.904	-155.479	-154.100	-155.229	-168.495	-172.727	-186.409
R^2	0.182	0.200	0.352	0.362	0.367	0.374	0.098	0.102	0.120	0.205	0.238	0.313

Note: Standardized coefficients reported. Robust standard errors reported in parenthesis * = significant at the 90% level, ** = significant at the 95% level, and *** = significant at the 99% level. ΔT represents the change in tokens collected between rounds 1-3 and rounds 4-6 (i.e. before and after the ecological change). HLr1 to HLr6 represent 6 regression equations estimated hierarchically, adding one new predictor for each equation, for the condition in which participants go from high to low resource growth rates. LH r1 to LHr6 represent the condition in which participants go from low to high resource growth rates.

Table 7: General Linear Model: Individual cognitive abilities and controlling factors on $Time$ using $avg(ToM)$ in groups

Dep.Var	HLst1	HLst2	HLst3	HLst4	HLst5	HLst6	LHst1	LHst2	LHst3	LHst4	LHst5	LHst6
$avg(ToM)$	0.081	0.091	0.231	-0.221	-1.084	-1.115	1.068	1.066	1.000	1.039	1.003	1.073
	(0.984)	(1.015)	(1.007)	(1.067)	(1.051)	(1.205)	(0.922)	(0.900)	(0.942)	(0.930)	(0.967)	(0.984)
$avg(g)$	0.114	0.118	0.212	0.030	-0.297	-0.312	0.197	0.196	0.150	0.192	0.176	0.205
	(0.396)	(0.406)	(0.410)	(0.435)	(0.435)	(0.508)	(0.369)	(0.360)	(0.388)	(0.381)	(0.394)	(0.402)
$avg(g) * avg(ToM)$	-0.025	-0.026	-0.033	-0.016	0.020	0.022	-0.036	-0.036	-0.032	-0.035	-0.031	-0.035
	(0.042)	(0.044)	(0.043)	(0.046)	(0.045)	(0.053)	(0.042)	(0.040)	(0.042)	(0.042)	(0.043)	(0.044)
Round 1 Baseline	0.000	0.000	0.000	0.000	0.000	0.000	0.000	0.000	0.000	0.000	0.000	0.000
	(.)	(.)	(.)	(.)	(.)	(.)	(.)	(.)	(.)	(.)	(.)	(.)
Round 2	-1.324***	-1.330***	-1.340***	-1.339***	-1.381***	-1.379***	-1.564***	-1.389***	-1.396***	-1.365***	-1.363***	-1.384***
	(0.466)	(0.469)	(0.469)	(0.472)	(0.442)	(0.444)	(0.458)	(0.486)	(0.483)	(0.486)	(0.482)	(0.486)
Round 3	-1.647***	-1.654***	-1.666***	-1.664***	-1.714***	-1.713***	-1.268***	-1.112**	-1.116***	-1.089**	-1.091**	-1.110**
	(0.493)	(0.532)	(0.532)	(0.526)	(0.471)	(0.471)	(0.410)	(0.433)	(0.431)	(0.436)	(0.447)	(0.457)
Round 4	-1.494***	-1.502***	-1.515***	-1.511***	-1.556***	-1.554***	-2.019***	-1.915***	-1.918***	-1.900***	-1.909***	-1.922***
	(0.427)	(0.434)	(0.444)	(0.448)	(0.413)	(0.410)	(0.432)	(0.445)	(0.446)	(0.448)	(0.440)	(0.446)
Round 5	-1.818***	-1.826***	-1.837***	-1.836***	-1.892***	-1.890***	-2.355***	-2.248***	-2.254***	-2.236***	-2.237***	-2.252***
	(0.401)	(0.420)	(0.422)	(0.424)	(0.385)	(0.384)	(0.444)	(0.460)	(0.459)	(0.465)	(0.466)	(0.471)
Round 6	-2.195***	-2.202***	-2.213***	-2.212***	-2.278***	-2.277***	-2.479***	-2.411***	-2.415***	-2.404***	-2.407***	-2.417***
	(0.392)	(0.418)	(0.424)	(0.430)	(0.428)	(0.428)	(0.464)	(0.465)	(0.467)	(0.471)	(0.476)	(0.482)
Chat		0.002	0.004	0.002	0.000	-0.000		-0.042	-0.041	-0.049	-0.051	-0.046
		(0.043)	(0.044)	(0.047)	(0.044)	(0.043)		(0.039)	(0.039)	(0.041)	(0.040)	(0.046)
Trust			-0.060	-0.052	0.067	0.069			0.039	0.032	0.063	0.071
			(0.083)	(0.080)	(0.097)	(0.095)			(0.092)	(0.090)	(0.094)	(0.100)
Religious Diversity				-0.507	-1.545***	-1.531***				0.268	-0.062	-0.066
				(0.348)	(0.446)	(0.533)				(0.371)	(0.488)	(0.493)
Ethnic Diversity					1.553***	1.544***					0.562	0.540
					(0.483)	(0.535)					(0.451)	(0.448)
Gender						-0.041						0.238
						(0.824)						(0.912)
Constant	1.472	1.368	-0.530	4.489	12.378	12.709	-6.896	-6.710	-5.771	-6.662	-6.722	-7.465
	(9.055)	(9.381)	(9.382)	(10.134)	(9.999)	(11.765)	(8.213)	(7.999)	(8.631)	(8.464)	(8.749)	(8.967)
N	162	162	162	162	162	162	150	150	150	150	150	150
AIC	108.753	110.752	112.602	114.050	112.794	114.793	124.691	126.241	128.168	129.998	131.383	133.354
D	0.244	0.246	0.247	0.245	0.224	0.226	0.364	0.363	0.365	0.367	0.365	0.367

Note: Robust standard errors reported in parenthesis * = significant at the 90% level, ** = significant at the 95% level, and *** = significant at the 99% level. Round 1 = baseline to which other rounds are compared to. HLr1 to HLr6 represent 6 regression equations estimated hierarchically, adding one new predictor for each equation, for the condition in which participants go from high to low resource growth rates. LH r1 to LHr6 represent the condition in which participants go from low to high resource growth rates.

Table 8: OLS Regression: Individual cognitive abilities and controlling factor on ΔT using $min(g)$ in groups

Dep. Var.	gHLsr1	gHLsr2	gHLsr3	gHLsr4	gHLsr5	gHLsr6	gLHsr1	gLHsr2	gLHsr3	gLHsr4	gLHsr5	gLHsr6
$min(ToM)$	0.017*	0.018*	0.034***	0.033***	0.036***	0.037***	0.076	0.064	0.008	0.042	0.155**	0.160***
	(0.010)	(0.010)	(0.008)	(0.008)	(0.010)	(0.010)	(0.063)	(0.061)	(0.051)	(0.052)	(0.067)	(0.057)
$min(g)$	0.011***	0.012***	0.022***	0.023***	0.024***	0.024***	0.038**	0.033*	0.001	0.005	0.044*	0.049**
	(0.004)	(0.004)	(0.004)	(0.004)	(0.005)	(0.005)	(0.019)	(0.019)	(0.016)	(0.016)	(0.022)	(0.020)
$min(g) * min(ToM)$	-0.002***	-0.002***	-0.003***	-0.003***	-0.003***	-0.003***	-0.005	-0.004	-0.001	-0.002	-0.009**	-0.010***
	(0.001)	(0.001)	(0.000)	(0.000)	(0.001)	(0.001)	(0.004)	(0.004)	(0.003)	(0.003)	(0.004)	(0.003)
$\Delta Chat$		-0.001	0.002	0.001	0.001	0.002		-0.006	-0.008	-0.004	-0.001	-0.001
		(0.001)	(0.001)	(0.001)	(0.002)	(0.002)		(0.005)	(0.006)	(0.004)	(0.004)	(0.004)
Trust			-0.014***	-0.013***	-0.012***	-0.012***			0.023***	0.025***	0.013	0.019**
			(0.002)	(0.002)	(0.003)	(0.003)			(0.007)	(0.008)	(0.008)	(0.008)
Religious Diversity				0.015	0.005	0.018				-0.136***	-0.038	-0.030
				(0.010)	(0.012)	(0.015)				(0.038)	(0.041)	(0.037)
Ethnic Diversity					0.016	0.008					-0.164***	-0.179***
					(0.014)	(0.015)					(0.034)	(0.036)
Gender						-0.035						0.219***
						(0.023)						(0.079)
Constant	0.078	0.068	-0.123*	-0.139**	-0.157**	-0.158**	-0.623*	-0.533	0.039	0.042	-0.612	-0.816**
	(0.071)	(0.071)	(0.068)	(0.069)	(0.079)	(0.079)	(0.336)	(0.326)	(0.285)	(0.282)	(0.390)	(0.356)
N	27	27	27	27	27	27	25	25	25	25	25	25
AIC	-489.138	-487.702	-515.572	-516.370	-515.663	-516.260	-148.815	-147.624	-153.182	-171.815	-189.111	-201.712
R^2	0.367	0.369	0.475	0.484	0.489	0.497	0.057	0.062	0.108	0.223	0.317	0.380

Note: Standardized coefficients reported. Robust standard errors reported in parenthesis * = significant at the 90% level, ** = significant at the 95% level, and *** = significant at the 99% level. ΔT represents the change in tokens collected between rounds 1-3 and rounds 4-6 (i.e. before and after the ecological change). HLr1 to HLr6 represent 6 regression equations estimated hierarchically, adding one new predictor for each equation, for the condition in which participants go from high to low resource growth rates. LH r1 to LHr6 represent the condition in which participants go from low to high resource growth rates.

Table 9: General Linear Model: Individual cognitive abilities and controlling factors on $Time$ using $min(g)$ in groups

Dep.Var	gHLst1	gHLst2	gHLst3	gHLst4	gHLst5	gHLst6	gLHst1	gLHst2	gLHst3	gLHst4	gLHst5	gLHst6
$min(ToM)$	0.288	0.288	0.181	0.290	0.622*	0.620*	0.946	0.906	1.187	1.069	0.915	0.928
	(0.323)	(0.323)	(0.353)	(0.344)	(0.320)	(0.326)	(0.719)	(0.724)	(0.759)	(0.745)	(0.877)	(0.878)
$min(g)$	0.026	0.026	-0.044	-0.020	0.115	0.119	0.283	0.273	0.454	0.476*	0.424	0.431
	(0.129)	(0.129)	(0.146)	(0.142)	(0.137)	(0.140)	(0.249)	(0.251)	(0.293)	(0.286)	(0.325)	(0.324)
$min(g) * min(ToM)$	-0.032*	-0.032*	-0.026	-0.033*	-0.053***	-0.053***	-0.052	-0.049	-0.066	-0.062	-0.052	-0.053
	(0.019)	(0.019)	(0.020)	(0.020)	(0.019)	(0.020)	(0.040)	(0.040)	(0.042)	(0.042)	(0.050)	(0.050)
Round 1 Baseline	0.000	0.000	0.000	0.000	0.000	0.000	0.000	0.000	0.000	0.000	0.000	0.000
	(.)	(.)	(.)	(.)	(.)	(.)	(.)	(.)	(.)	(.)	(.)	(.)
Round 2	-1.392***	-1.411***	-1.411***	-1.429***	-1.517***	-1.530***	-1.500***	-1.352***	-1.342***	-1.262**	-1.251**	-1.268**
	(0.435)	(0.441)	(0.435)	(0.440)	(0.416)	(0.417)	(0.453)	(0.484)	(0.489)	(0.495)	(0.493)	(0.494)
Round 3	-1.727***	-1.750***	-1.751***	-1.773***	-1.878***	-1.894***	-1.214***	-1.080**	-1.074**	-1.000**	-0.992**	-1.008**
	(0.451)	(0.478)	(0.469)	(0.471)	(0.442)	(0.439)	(0.416)	(0.442)	(0.450)	(0.454)	(0.456)	(0.463)
Round 4	-1.568***	-1.593***	-1.593***	-1.617***	-1.722***	-1.739***	-1.941***	-1.855***	-1.861***	-1.825***	-1.823***	-1.833***
	(0.456)	(0.449)	(0.443)	(0.454)	(0.431)	(0.417)	(0.453)	(0.464)	(0.466)	(0.470)	(0.465)	(0.472)
Round 5	-1.905***	-1.928***	-1.930***	-1.952***	-2.069***	-2.087***	-2.269***	-2.182***	-2.186***	-2.152***	-2.145***	-2.157***
	(0.401)	(0.414)	(0.411)	(0.417)	(0.393)	(0.388)	(0.461)	(0.469)	(0.471)	(0.480)	(0.479)	(0.484)
Round 6	-2.293***	-2.314***	-2.317***	-2.336***	-2.454***	-2.471***	-2.391***	-2.347***	-2.359***	-2.351***	-2.346***	-2.351***
	(0.438)	(0.467)	(0.467)	(0.471)	(0.461)	(0.461)	(0.512)	(0.512)	(0.504)	(0.511)	(0.513)	(0.519)
Chat		0.005	0.004	0.009	0.020	0.024		-0.036	-0.040	-0.064	-0.066	-0.063
		(0.041)	(0.039)	(0.039)	(0.036)	(0.035)		(0.038)	(0.038)	(0.040)	(0.041)	(0.046)
Trust			0.098	0.104	0.218**	0.212**			-0.153	-0.187*	-0.170	-0.165
			(0.088)	(0.085)	(0.091)	(0.090)			(0.101)	(0.109)	(0.120)	(0.128)
Religious Diversity				-0.364	-1.380***	-1.569***				0.803**	0.664	0.664
				(0.320)	(0.432)	(0.556)				(0.389)	(0.498)	(0.496)
Ethnic Diversity					1.536***	1.637***					0.258	0.242
					(0.421)	(0.499)					(0.522)	(0.524)
Gender						0.437						0.196
						(0.705)						(0.969)
Constant	1.025	0.996	2.287	2.232	0.098	-0.079	-5.239	-4.920	-8.171	-8.892*	-8.049	-8.270
	(2.091)	(2.110)	(2.388)	(2.310)	(2.173)	(2.265)	(4.546)	(4.558)	(5.300)	(5.151)	(5.803)	(5.768)
N	162	162	162	162	162	162	150	150	150	150	150	150
AIC	103.909	105.903	107.509	109.230	108.075	109.975	128.960	130.622	131.715	132.146	134.028	136.007
D	0.213	0.214	0.213	0.212	0.193	0.193	0.394	0.395	0.391	0.382	0.384	0.387

Note: Robust standard errors reported in parenthesis * = significant at the 90% level, ** = significant at the 95% level, and *** = significant at the 99% level. Round 1 = base round to which other rounds are compared to. HLr1 to HLr6 represent 6 regression equations estimated hierarchically, adding one new predictor for each equation, for the condition in which participants go from high to low resource growth rates. LH r1 to LHr6 represent the condition in which participants go from low to high resource growth rates.

7 Supplementary Method 5

7.1 Resource Experiment Design

This experiment is focused on understanding how social and general intelligence affect group performance under changing environmental conditions. The experiment is based on the work of Janssen and colleagues [12]

The software used for this experiment is open-source and available at http://commons.asu.edu. However, the actual software has been modified to fit our experimental needs and modifications will be made publicly available in the near future.

To participate in our experiments, individuals need to release their ACT/SAT scores and perform a social intelligence test (see below). In the actual experiment, individuals are randomly assigned to groups of 4 and need to harvest tokens in a 20X20 grid. ACT/SAT scores served as our measure and proxy of g, and the short story test served as our measure and proxy of ToM.

Each token harvest is equal to $0.02. Participants can see the whole grid and thus have information on how other individuals within their own group behave. However, they have no information on other groups participating in the experimental session. Further, participants are able to communicate with other group members between rounds and before the 1st round. Each round last 180 seconds and at the beginning of each round tokens fill 15% of the grid. Empty cells can generate token with probability $p_{tok} = p_g * n_{tok}/N$ where p_g is the maximum growth rate (= 0.01 in case of high growth rate, and 0.005 in case of low growth rate), n_{tok} = the number of neighboring cells with tokens and N = 8 representing the maximum number of neighboring cells that can have tokens. In other words, the more tokens are neighboring an empty cell, the higher the probability that the empty cell will generate tokens. If all neighboring cells are empty, no token will grow. Growth rate was always changed after round three, hence participants played three rounds with either low or high growth rate, and three rounds with high

or low growth rate.

8 Supplementary Method 6

8.1 Recruitment for the Experiments

We recruited 216 undergraduate students from two universities: Utah State University and the University of Texas at San Antonio. We recruited students at UTSA from the introductory psychology participation pool. The introductory psychology participation pool draws students from approximately 8 introductory psychology courses at over 100 students per course where students complete studies for partial credit toward course completion. These intro courses draw students from across the university. At USU, we recruited students from introductory sociology and anthropology courses that have over a 150 students enrolled per course. These courses are general requirement courses and draw students from all majors represented at the university.

Participation in the study was voluntary and students could withdraw participation at any time.

9 Supplementary Method 7

9.1 Experiment Protocol

To facilitate the replication of this work we provide the code used to run the experiment here: https://bitbucket.org/tamnguyenthe/fip-game We also provide a video of a round of the experimental session as a supplementary file.

Once individuals were seated at their experimental stations, the following instructions where given:

Welcome to the Packman Game. You have completed the consent form, and you are now ready to participate. Please give your best effort. You will receive academic credit for partici-

pating. In addition, based on your performance, you can also earn up to $40. The experiment will take 1 to 1.5 hours, and is dived into three parts. In Part I you will complete an ”eyes task”. In Part II, you will play a foraging game. In Part III you will complete a ”short story task” and answer a few questions on an exit survey. Please no talking during the experimental session. You must have completed the ACT or SAT and be 18 years or older to participate. Thank you for your participation!

No questions from participants were allowed during the experiment to minimize facilitator interference.

9.2 Resource Growth Change Experiment

Following are the instructions, as they appeared on screen before the actual token harvest experiment:

In this game you will earn money for collecting tokens. The amount of money you earn depends on your decisions AND the decisions of other people in this room over the course of playing a game described below.

How to play

You will appear on the screen as a yellow dot (avatar) with other individuals who will appear as avatars. You can move by pressing the four arrow keys on your keyboard.

You can move up, down, left, or right. You have to press a key for each and every move of your yellow dot. As you move around you can collect green diamond shaped tokens and earn two cents for each collected token. To collect a token, move your yellow dot over a green token and press the space bar. Simply moving your avatar over a token does NOT collect that token.

Between rounds of token collecting you will have 1 minute to chat via text box.

Tokens

The tokens that you collect have the potential to regenerate. After you have collected a

green token, a new token can re-appear on that empty cell. The rate at which new tokens appear depends on the number of adjacent cells with tokens. The more tokens in the eight cells that surround an empty cell, the faster a new token will appear on that empty cell. Existing tokens can generate new tokens. To illustrate this, please refer to Image 1 and Image 2. The middle cell in Image 1 denoted with an X has a greater chance of regeneration than the middle cell in Image 2. When all neighboring cells are empty, there is no chance for regeneration.

Best Strategy

The chance that a token will regenerate on an empty cell increases as there are more tokens surrounding it. Therefore, you want to have as many tokens around an empty cell as possible. However, you also need empty cells to benefit from this regrowth. The best arrangement of tokens that maximizes overall regrowth is the checkerboard diagram shown below. The slower the token regrowth, the more patient you must be in order for a token to reappear after harvest.

10 Supplementary Method 8

Short Story Test

In the Short Story Task (SST), participants read ”The End of Something”, a short story by Ernest Hemingway, which presents a nuanced interaction between a romantic couple in which the male protagonist, Nick, starts an argument and breaks up with his girlfriend, Marjorie. Through the course of the story, the characters display sarcasm, non-verbal and indirect communication, higher-order emotions like guilt, and attempts to hide their intentions and feelings from one another.

According to Dodell-Feder [2], the goal of the SST was to

> ”to design a new ToM task (the Short Story Task -SST-) that improved upon the limitations of existing ToM measures. More specifically, we aimed to create a task

that (a) was sensitive to individual differences in ToM ability and did not suffer from ceiling effects, (b) incorporated a range of mental states of differing complexity, including epistemic states, affective states, and intentions to be inferred from a first- and second-order level, (c) used ToM stimuli representative of real-world social interactions, (d) required participants to utilize social context when making mental state inferences, (e) exhibited adequate psychometric properties, and (f) was quick and easy to administer and score." [2, p. 2]

10.0.1 Short Story Test instruction and questions

Instructions to Participant

Now you are going to read a short story called The End of Something. The story is only a few pages, but take your time reading it. Try to get a sense of what happens and what the relationships are between the characters. After you're finished, some questions will appear on the screen and you will be asked to answer them.

After story is read

1. Have you read this story before? [yes — no]
   - IF YES
     - How long ago did you read it?
     - How well do you remember the story?
     - Did you read it for school or pleasure?
       - IF SCHOOL
         - What grade were you in?
         - What class was it for?
2. Is the story familiar to you? [yes — no]

- IF YES
    - Do you know anything about the story? What do you know about it?
    - Have you discussed the story with anyone?

Instructions to Participant Now I'm going to ask you some questions about the story. Here is a copy of the questions I'll be asking so you can read along. For most of the questions, there are no right or wrong answers and the questions can be answered with short responses. We're also interested in the character's thoughts, feelings and intentions when it applies to the question.

Questions

1. In just a few sentences, how would you summarize the story
2. What do Nick and Marjorie observe on the shoreline as they are rowing to the point to set their fishing lines?
3. What does Nick mean when he says, ”They aren't striking?”
4. Nick and Marjorie have a pail of perch for what purpose?
5. Do Marjorie's actions suggest that she is experienced or inexperienced at fishing? What makes you say that?
6. Why does Nick say to Marjorie, ”You know everything”?
7. Why does Marjorie reply, ”Oh Nick, please cut it out! Please, please don't be that way!”?
8. Why is Nick afraid to look at Marjorie?
9. What does Nick mean when he says, ”It isn't fun anymore”?

10. Why does Marjorie sit with her back toward Nick when she asks, ”Isn’t love any fun?”?

11. Why does Marjorie take the boat and leave and what is she feeling at that moment?

12. Who is Bill and what does he reveal when he asks Nick, ”Did she go alright? ... Have a scene?”?

13. What is Nick feeling when he says, ”Oh, go away, Bill! Go away for a while”?

14. The story is called ”The End of Something.” What is the title referring to?

10.0.2 Scoring the Short Story Test

Scoring for the SST according to [29]. Three different coders coded the answer to the SST independently. We calculated Krippendorff’s alpha using ordinal data using ReCal online. Krippendorff’s alpha (ordinal) was 0.833, demonstrating a high level of coders agreement. As the SST was consistently coded with three coders, all questions that did not have 100% agreement among coders was coded as the score issued by the majority of coders. If a question did not have a majority (all coders issued different scores) disagreements were resolved via discussion between all coders.

The following is the coding sheet used by the coders. Explicit mental state reasoning (in bold) is the metric used to assess Social Intelligence.

- Comprehension: Sum scores of 5 comprehension questions (questions 2, 3, 4, 5, and 14). Ranges from 0 to 10.

- **Explicit mental state reasoning**: Sum scores of 8 mental state reasoning questions (questions 6, 7, 8, 9, 10, 11, 12, and 13). Ranges from 0 to 16.

- Spontaneous mental state inference: 1 score for spontaneous mental state question (question 1). Ranges from 0 to 1.

Following are examples of coding used to evaluate and score the three components of the test described above.

- Question 1: 1 = any mental state inference, even if it is wrong
- Question 2: 2 = any adjective + mill 1 = only mill 0 = anything else
- Question 5: 2 = experienced 2 or 1 = somewhat experienced / somewhat inexperienced 2 for good justification 1 for bad / no justification 1 or 0 = inexperienced 1 for good justification 0 for bad / no justification
- Question 6: 0 = anything that does not understand that he's being sarcastic, anything that thinks he's joking, anything that thinks that she does actually know everything
- Question 7: 2 = if they understood that he was giving her a hard time or doing something that was not intended to make her happy
- Question 8: 2 = anything that references her reaction / emotions 1 = anything that references his reaction / emotions without referencing hers 0 = no mention of an emotion
- Question 10: 2 = knows about break up / something bad (may include emotion) 1 = emotion with no knowledge of breakup / something bad 0 = No emotion, No knowledge about break up / something bad
- Question 11: 2 = (either the relationship is over or wanting space) AND negative emotion 1= upset OR wants space
- Question 12: 2 = Bill's relationship with Nick AND anything that references Bill's advanced knowledge 1 = Bill's relationship with Nick OR directly states Bill knew Nick was going to break up with Marjorie (Bill is not in the clearing while Nick and Marjorie fight and/or break up. He enters later.)

- Question 13: 2 = negative emotion referencing break up AND needs space / doesn't want to talk 1 = negative emotion 0 = no negative emotion, only wants space

- Miscellaneous: As long as a correct answer is present (even if a patently wrong answer is also present), give the score for the correct answer. Anything that is obviously wrong (outside of question 1) should be scored as 0.

11 Supplementary Method 9

11.1 Survey

Following is the exit survey.

1. Please report your age in years.

2. Please provide your current GPA

3. Please describe your religious affiliation, if any. Please be as specific as possible.

4. What is your primary language?

5. Please specify how you identify your race or ethnicity.

6. Please indicate the number of individuals who you call close friends? Please exclude family members and mere acquaintances.

7. How many individuals are in your total social network (i.e., close friends plus family members plus acquaintances)?

8. Please write the typical number of individuals who lived in your home while you were between the ages of 5-17?

9. Please estimate the median household income of the family in which you lived between ages of 5-17?
10. What is your college major or intended college major?
11. Please circle the descriptor that best describes your biological sex?
    - M
    - F
12. Did you understand the instructions of the exercises?
    - I did not understand anything I understood only a bit of the instructions
    - I understood half of the instructions
    - I understood most of the instructions
    - I understood everything
13. Do you think most people would try to take advantage of you if they had a chance, or would they try to be fair?
    - Would take advantage of you
    - Depends on situation
    - Would try to be fair
14. Would you say that most of the time people try to be helpful, or that they are mostly just looking out for themselves?
    - Try to be helpful
    - Depends on situation

- Mostly just looking out for themselves

15. Generally speaking, would you say that most people can be trusted or that you can't be too careful in dealing with people?

    - Most people can be trusted
    - Depends on situation
    - Can't be too careful in dealing with people

16. In the past year, did you do any volunteer activity through organizations; i.e. donate your time and energy not for pay?

    - Yes
    - No

17. Global warming is a fact and is mostly caused by emissions from vehicles and industrial facilities?

    - I completely agree
    - I somewhat agree
    - I have no opinion
    - I somewhat disagree
    - I completely disagree

18. Tell me whether the first statement or the second statement comes closer to your own views ? even if neither is exactly right.

    - Most people who want to get ahead can make it if they're willing to work hard.

- Hard work and determination are no guarantee of success for most people.

19. Tell me whether the first statement or the second statement comes closer to your own views ? even if neither is exactly right.
    - The government should do more to help needy Americans, even if it means going deeper into debt.
    - The government today can't afford to do much more to help the needy
20. Here are two statements people sometimes make when discussing the environment and economic growth. Which of them comes closer to your own point of view?
    - Protecting the environment should be given priority, even if it causes slower economic growth and some loss of jobs.
    - Economic growth and creating jobs should be the top priority, even if the environment suffers to some extent.
21. What is the highest educational level that your Parents have attained?
22. What is your Father's occupation?
23. What is your Mother's occupation?

12 Supplementary References

References

[1] Coyle, T. R. & Pillow, D. R. Sat and act predict college gpa after removing g. *Intelligence* **36**, 719–729 (2008).

[2] Dodell-Feder, D., Lincoln, S. H., Coulson, J. P. & Hooker, C. I. Using fiction to assess mental state understanding: A new task for assessing theory of mind in adults. *PLoS ONE* **8**, 1–14 (2013).

[3] Hardin, G. The tragedy of the commons. *Science* **162**, 1243–48 (1968).

[4] Berkes, F., Feeny, D., McCay, B. J. & Acheson, J. M. The benefits of the commons. *Nature* **340**, 91 (1989).

[5] Dietz, T. Struggle to Govern the Commons. *Science* **302**, 1907–1912 (2003).

[6] Ostrom, E. *Governing the commons: The evolution of institutions for collective action* (Cambridge University Press, 1990).

[7] Baggio, J. A. *et al.* Explaining success and failure in the commons: the configural nature of Ostrom's institutional design principles. *International Journal of the Commons* **10**, 417–439 (2016).

[8] Barnett, A. *et al.* An iterative approach to case study analysis: Insights from qualitative analysis of quantitative inconsistencies. *International Journal of the Commons* **10** (2016).

[9] Cox, M., Arnold, G. & Tomás, S. V. A Review of Design Principles for Community-based Natural Resource. *Ecology and Society* **15**, 38 (2010).

[10] Freeman, J., Coyle, T. R. & Baggio, J. A. The functional intelligences proposition. *Personality and Individual Differences* **99**, 46–55 (2016).

[11] Nettle, D. & Liddle, B. Agreeableness is related to social-cognitive, but not social-perceptual, theory of mind. *European Journal of Personality* **22**, 323–335 (2008).

[12] Janssen, M. A., Holahan, R., Lee, A. & Ostrom, E. Lab experiments for the study of social-ecological systems. *Science* **328**, 613–617 (2010).
