# Supplementary material for: The importance of cognitive diversity for sustaining the commons
Source: Nat Commun. 2019 Feb 20;10:875. doi: 10.1038/s41467-019-08549-8 (PMC6382855; doi:10.1038/s41467-019-08549-8)
Supplement: Supplementary file 3 — Description of Additional Supplementary Files [file 41467_2019_8549_MOESM3_ESM.docx]

**Description of Data**

File Name: Data 1

Description: The dataset contains values for the variable used in the analysis. Both the main variables of interest *g* and *ToM* as well as the controlling variables used (ethnic and religious diversity, chat volume, trust, and gender, as well as the basic performance measures (*time* -timeleft- and token collected per round as a % of maximum possible token that could have been collected -tokper-). Both *avg T* and *Delta T* are calculated based on tokper (see Methods and Supplementary Method 1.
